# Supplementary material for: Ebola virus matrix protein VP40 triggers inflammatory responses linked to the ebolavirus virulence
Source: Proc Natl Acad Sci U S A. 2025 Dec 29;123(1):e2508194123. doi: 10.1073/pnas.2508194123 (PMC12773709; doi:10.1073/pnas.2508194123)
Supplement: Supplementary file 1 — Appendix 01 (PDF) [file pnas.2508194123.sapp.pdf]

PNAS

Supporting Information for

Ebola Virus Matrix Protein VP40 Triggers Inflammatory Responses Linked to the Ebolavirus Virulence

Satoko Yamaoka[1,†], Zeineb M'Hamdi[2], Lin Wang[1], Vaille A. Swenson[3], Kristin L. McNally[4], Shao-Chia Lu[1], Reema Singh[2], Stephanie L. Saundh[2], Brady N. Zell[3], Sonja M. Best[4,5], Michael A. Barry[1,6], Angela L. Rasmussen[2,7,8], Hideki Ebihara[9*]

[1]Department of Medicine, Division of Infectious Diseases, Mayo Clinic, Rochester, MN 55905, USA

[2]Vaccine and Infectious Disease Organization, University of Saskatchewan, Saskatoon, SK S7N 5E3, Canada

[3]Virology and Gene Therapy Track, Mayo Graduate School of Biomedical Sciences, Rochester, MN 55905, USA

[4]Laboratory of Virology, Rocky Mountain Laboratories, National Institute of Allergy and Infectious Diseases, NIH, Hamilton, MT 59840, USA

[5]Laboratory of Neurological Infections and Immunity, Rocky Mountain Laboratories, National Institute of Allergy and Infectious Diseases, NIH, Hamilton, MT 59840, USA

[6]Department of Immunology, Mayo Clinic, Rochester, MN 55905, USA

[7]Department of Biochemistry, Microbiology, and Immunology, University of Saskatchewan, Saskatoon, SK S7N 5E3, Canada

[8]Department of Ecology and Evolution, Stony Brook University, Stony Brook, NY 11794, USA

[9]Department of Virology 1, National Institute of Infectious Diseases, Japan Institute for Health Security, Tokyo 162-8640, Japan

[†]Present address: HDT Bio, Seattle, WA 98109, USA.

*Corresponding author: Hideki Ebihara (hebihara@niid.go.jp)

This PDF file includes:

Supporting text

Figures S1 to S13

Figure legends for S1 to S13

Tables S1 to S3

SI References

Other supporting materials for this manuscript include the following:

Datasets S1 to S3

Supporting information text

Materials and Methods

Plasmids

EBOV (variant Mayinga) protein expression plasmids pCAGGs-NP, -VP35, -VP40, -GP, -VP30, -VP24 and -L were previously generated (1, 2) utilizing a pCAGGs vector possessing the cytomegalovirus enhancer fused to the chicken beta-actin promoter (3). These EBOV protein-expression plasmids have been routinely used in our lab for recombinant EBOV rescue (1) and in EBOV life cycle modeling systems (e.g., minigenomes and transcription and replication competent virus-like particles [trVLPs]) (4), and the expressed proteins are known to be functional. Additionally, VP40 derived from various EBOV variants (variant Kikwit and Makona-C07), as well as N-terminally FLAG-tagged VP40 from EBOV (variant Mayinga), SUDV (variant Gulu), BDBV, TAFV, and RESTV (variant Pennsylvania), and TRAF6 were cloned into a pCAGGs vector using standard cloning techniques.

A pNFκB-luc (AF053315.1) encoding a firefly luciferase gene driven by five copies of NF-κB response elements positioned upstream of a minimal promoter, and a pRL-TK (Promega) encoding a *Renilla* luciferase gene under the control of HSV-thymidine kinase promoter were utilized for dual-luciferase reporter (DLR) assays.

The following open reading frames were cloned into an expression vector under the control of the phosphoglycerate kinase promoter: IKK-2 and its mutant IKK-2 K44M (Addgene plasmids #11103 and #11104, gifts from Anjana Rao (5)); 3xHA-Iκβα and its mutants 3xHA-Iκβα-SS32/36AA (Addgene plasmids #21985 and #24143, gifts from Warner Greene (6)); TNFR1-YFP (Addgene plasmid #111209, gift from Johannes A. Schmid).

Single guide RNA (gRNA) (**SI Appendix, Table S1**) were cloned into a lentiCRISPR v2 (Addgene plasmid #52961, a gift from Feng Zhang (7)).

Gene knockout by CRISPR-Cas9

After limiting dilution of cells transfected with lentiCRISPR v2 encoding gRNA, genomic DNA was extracted from each cell line using QIamp DNA Mini Kit (Qiagen). The extracted DNA was then utilized as the template for PCR amplification, generating a fragment of approximately 300 bp that includes CRISPR editing site. PCR amplicon was employed for Sanger sequencing, and CRISPR editing were determined by using the Synthego ICE Analysis tool v3.0, western blotting, and flow cytometry (**Fig 6b, SI Appendix, Fig. S12, S13**).

qRT-PCR

Each reaction used 0.1 µg of RNA, 0.2 µM of forward and reverse primers, and 0.1 µM or 0.05 µM of probe with 5' 6FAM fluorophore and 3' BlackBerry quencher (BBQ) (TIB Molbiol) for target cytokine RNA and control GAPDH RNA, respectively. The primer/probe sequences are shown in the **SI Appendix, Table S3**. Cycling conditions were as follows: 10 min initial reverse transcription at 50°C, 2 min initial denaturation/activation at 95°C, followed by 40 cycles of 5 sec denaturation at 95°C and 10 sec annealing/extension step. Delta-delta C_t values were used to determine their relative expression as fold changes. qPCR was performed using the CFX384 Touch Real-Time PCR Detection System (BioRad), and data were analyzed with BioRad CFX Manager 3.1.

RNA-seq and transcriptomic analysis

RNA quality control was performed using an Agilent Bioanalyzer 2100 and samples were sent to the Saskatchewan Global Institute for Food Security (GIFS), where library prep was performed using the Illumina TruSeq Stranded mRNA Library kit and libraries were run on an Illumina NovaSeq6000.

Alignments and differential expression analysis were performed using an established pipeline. The sequence read quality of all 36 samples was examined using

Fastqc v0.11.9 (8). The paired-end fastq reads were mapped to the human reference genome assembly GRCh38 (9) (ensemble release-109) using STAR v2.7.10b (10). The genome index was generated using parameter "--sjdbOverhang 150" in STAR using reference genome and its annotation in GTF format. The raw counts were generated from the alignment files using the Rsubread v2.12.3 Bioconductor package (11) in R v4.2.2. The resulting read counts were imported in R and genes were filtered based on expression level (to keep only rows that have a count of 15 or higher in at least 6 samples) and differential expression analysis was performed using Bioconductor package DESeq2 v1.38.3 (12). Differentially expressed genes (DEGs) were identified using Wald test in DESeq2. To control the false discovery rate (FDR), p values were adjusted by applying the Benjamini-Hochberg (BH) method at an FDR cut-off of 5% (i.e., alpha=0.05) during differential expression analysis. Multidimensional scaling (MDS) plot of differentially expressed genes (filtered using adjusted P-value <0.01 and log2 fold change >1.5 and -1.5) was generated in R using ggplot2, vsn, and tidyverse packages.

For functional analysis, DEGs were uploaded to IPA (QIAGEN Bioinformatics), a manually curated database of experimentally demonstrated intermolecular relationships from the peer-reviewed literature for human, rat, and mouse experimental systems. The IPA Knowledgebase is updated on a quarterly basis and these analyses were performed with the most recent update (spring 2024) at the time of writing. The thresholds (fold change relative to time-matched, mock-infected controls > |1.5|, adjusted P-value < 0.05) were applied prior to running IPA Core Analysis.

Gene set enrichment analysis (GSEA) was used to analyze DEGs using hallmark gene sets available from the most recent release of the Human Molecular Signatures Database (MSigDB) (13) and clusterProfiler (14) v4.10.1 in R v4.3.3 (15). The thresholds (fold change relative to time-matched, mock-infected controls > |1.5| (log2FC > |0.585|), adjusted P-value < 0.05) were applied prior to GSEA as described above. Pathways that contained 10 or more DEGs were selected for analysis. Dot plots showing clusterProfiler enrichment results from hallmark gene sets were generated using enrichplot (16). Heatmaps were generated using pheatmap (17) v1.0.12 in R v.4.3.3. Volcano plots representing all identified DEGs were generated using ggplot2 (18) and R v4.3.3.

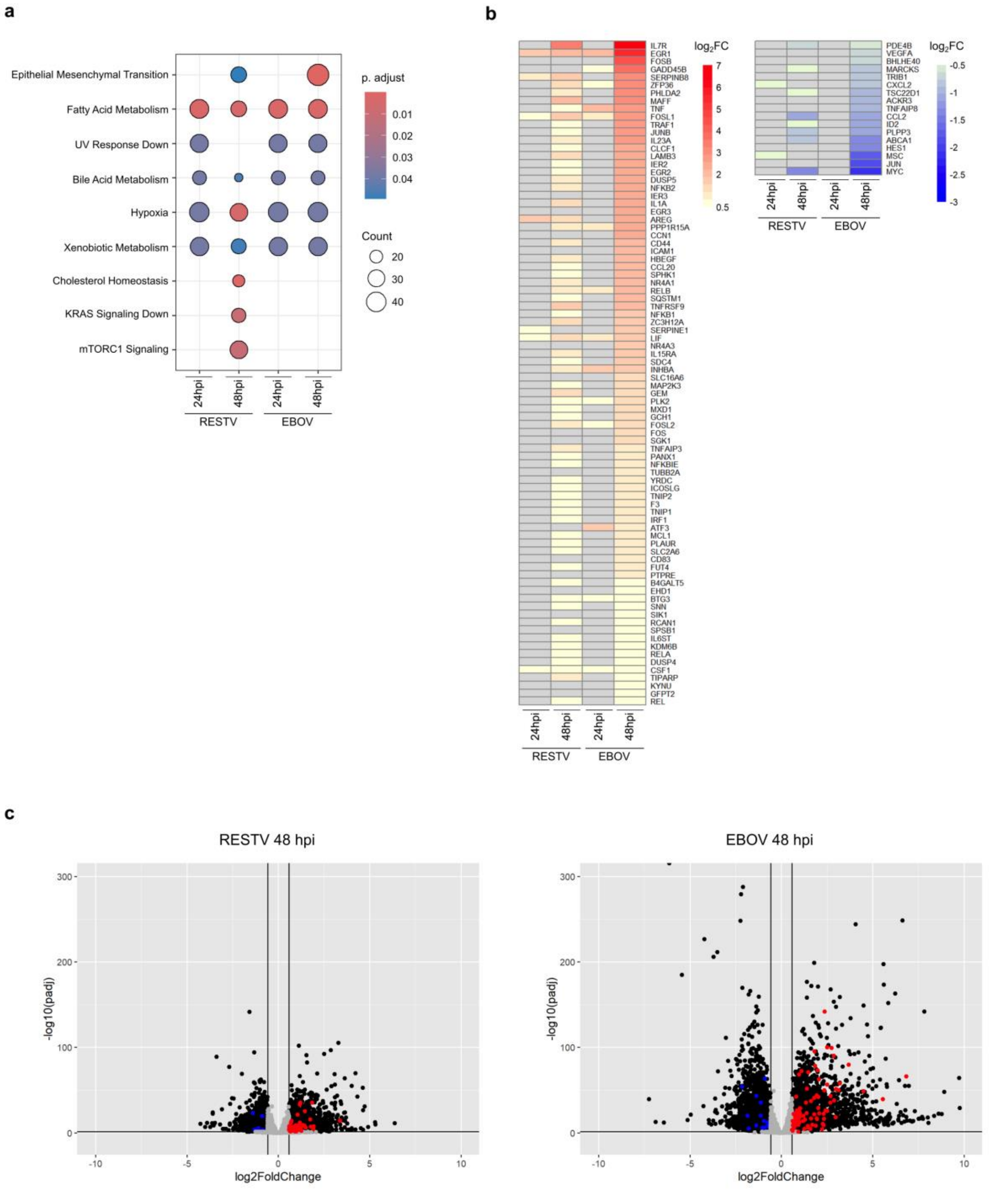

Supplementary Fig. 1: Transcriptomic analyses of Hallmark 'TNFA Signaling via NFKB' gene set using RNA-seq results from EBOV, or RESTV-infected 293 cells. a Dot plot showing GSEA hallmark pathways that contained 10 or more downregulated DEGs. Gene sets were sorted to only include genes that reached criteria (fold change relative to time-matched, mock-infected controls < 1.5, adjusted *P*-value < 0.05) within the

EBOV 48 hpi dataset. Further details on GSEA are provided in the Materials and Methods section. **b** Heatmaps for upregulated DEGs (left) and downregulated DEGs (right) generated using the Hallmark 'TNFA Signaling via NFKB' (M5890) (16) gene set available from MSigDB. Gene sets were sorted to only include genes that reached criteria (fold change relative to time-matched, mock infected controls > |1.5| [$\log_2$FC > |0.585|], adjusted *P*-value < 0.05) within the EBOV 48 hpi dataset. Gray color indicates genes that did not reach DE criteria in given dataset. Heatmaps were generated using the R package pheatmap v1.0.12 (17). **c** Volcano plots showing DEGs in RESTV and EBOV 48 hpi datasets. Red dots indicate upregulated and blue dots indicate downregulated DEGs within the 'TNFα Signaling via NF-κB' GSEA Hallmark pathway. Gray dots indicate genes that did not meet the DE criteria. Black dots indicate all other DEGs not within the 'TNFα Signaling via NF-κB' GSEA Hallmark pathway. Vertical lines indicate $\log_2$FC cutoffs, horizontal line indicates adjusted *P*-value cutoff. Plots were generated using the R package ggplot2 (18) v3.5.1.

a

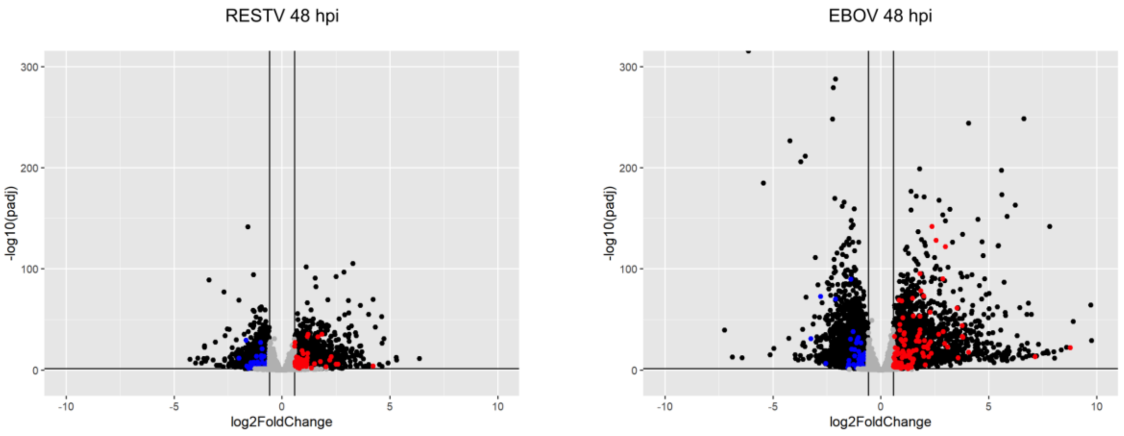
RESTV 48 hpi
EBOV 48 hpi
-log10(padj)
log2FoldChange
300
200
100
0
-10
-5
0
5
10

b

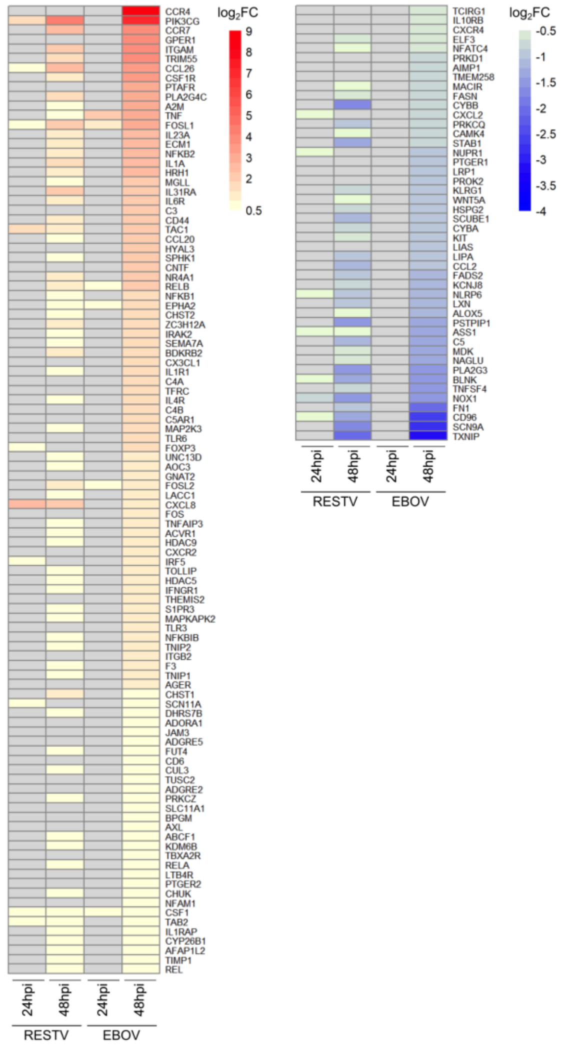
log_2FC
9
8
7
6
5
4
3
2
0.5
CCR4
PIK3CG
CCR7
GPER1
ITGAM
TRIM55
CCL26
CSF1R
PTAFR
PLA2G4C
A2M
TNF
FOSL1
IL23A
ECM1
NFKB2
IL1A
HRH1
MGLL
IL31RA
IL6R
C3
CD44
TAC1
CCL20
HYAL3
SPHK1
CNTF
NR4A1
RELB
NFKB1
EPHA2
CHST2
ZC3H12A
IRAK2
SEMA7A
BDKRB2
CX3CL1
IL1R1
C4A
TFRC
IL4R
C4B
C5AR1
MAP2K3
TLR6
FOXP3
UNC13D
AOC3
GNAT2
FOSL2
LACC1
CXCL8
FOS
TNFAIP3
ACVR1
HDAC9
CXCR2
IRF5
TOLLIP
HDAC5
IFNGR1
THEMIS2
S1PR3
MAPKAPK2
TLR3
NFKBIB
TNIP2
ITGB2
F3
TNIP1
AGER
CHST1
SCN11A
DHRS7B
ADORA1
JAM3
ADGRE5
FUT4
CD6
CUL3
TUSC2
ADGRE2
PRKCZ
SLC11A1
BPGM
AXL
ABCF1
KDM6B
TBXA2R
RELA
LTB4R
PTGER2
CHUK
NFAM1
CSF1
TAB2
IL1RAP
CYP26B1
AFAP1L2
TIMP1
REL
24hpi
48hpi
24hpi
48hpi
RESTV
EBOV
log_2FC
-0.5
-1
-1.5
-2
-2.5
-3
-3.5
-4
TCIRG1
IL10RB
CXCR4
ELF3
NFATC4
PRKD1
AIMP1
TMEM258
MACIR
FASN
CYBB
CXCL2
PRKCQ
CAMK4
STAB1
NUPR1
PTGER1
LRP1
PROK2
KLRG1
WNT5A
HSPG2
SCUBE1
CYBA
KIT
LIAS
LIPA
CCL2
FADS2
KCNJ8
NLRP6
LXN
ALOX5
PSTPIP1
ASS1
C5
MDK
NAGLU
PLA2G3
BLNK
TNFSF4
NOX1
FN1
CD96
SCN9A
TXNIP
24hpi
48hpi
24hpi
48hpi
RESTV
EBOV

Supplementary Fig. 2: Transcriptomic analyses of 'Inflammatory Response (GO:0006954)' gene set from AmiGO 2 using RNA-seq results from EBOV- or RESTV-infected 293 cells. a Volcano plots. Red dots indicate upregulated and blue dots indicate downregulated DEGs within the 'Inflammatory Response (GO:0006954)' from AmiGO 2 sorted to include only protein coding transcripts from *Homo sapiens*. Gray dots indicate genes that did not meet the DE criteria. Black dots indicate all other DEGs not within the 'Inflammatory Response (GO:0006954)' gene set. Vertical lines indicate $\log_2$FC cutoffs, horizontal line indicates adjusted *P*-value cutoff. Plots were generated using the R package ggplot2 (18) v3.5.1. **b** Heatmaps for upregulated DE genes (left) and downregulated DE genes (right) generated using the 'Inflammatory Response (GO:0006954)' gene set. Gene sets sorted to only include genes that reached criteria (absolute fold change relative to time-matched, mock infected controls > 1.5 [log2FC > 0.585], adjusted *P*-value < 0.05) within the EBOV 48 hpi dataset. Grey color indicates genes that did not reach DE criteria in given dataset. Heatmaps were generated using the R package pheatmap v1.0.12 (17).

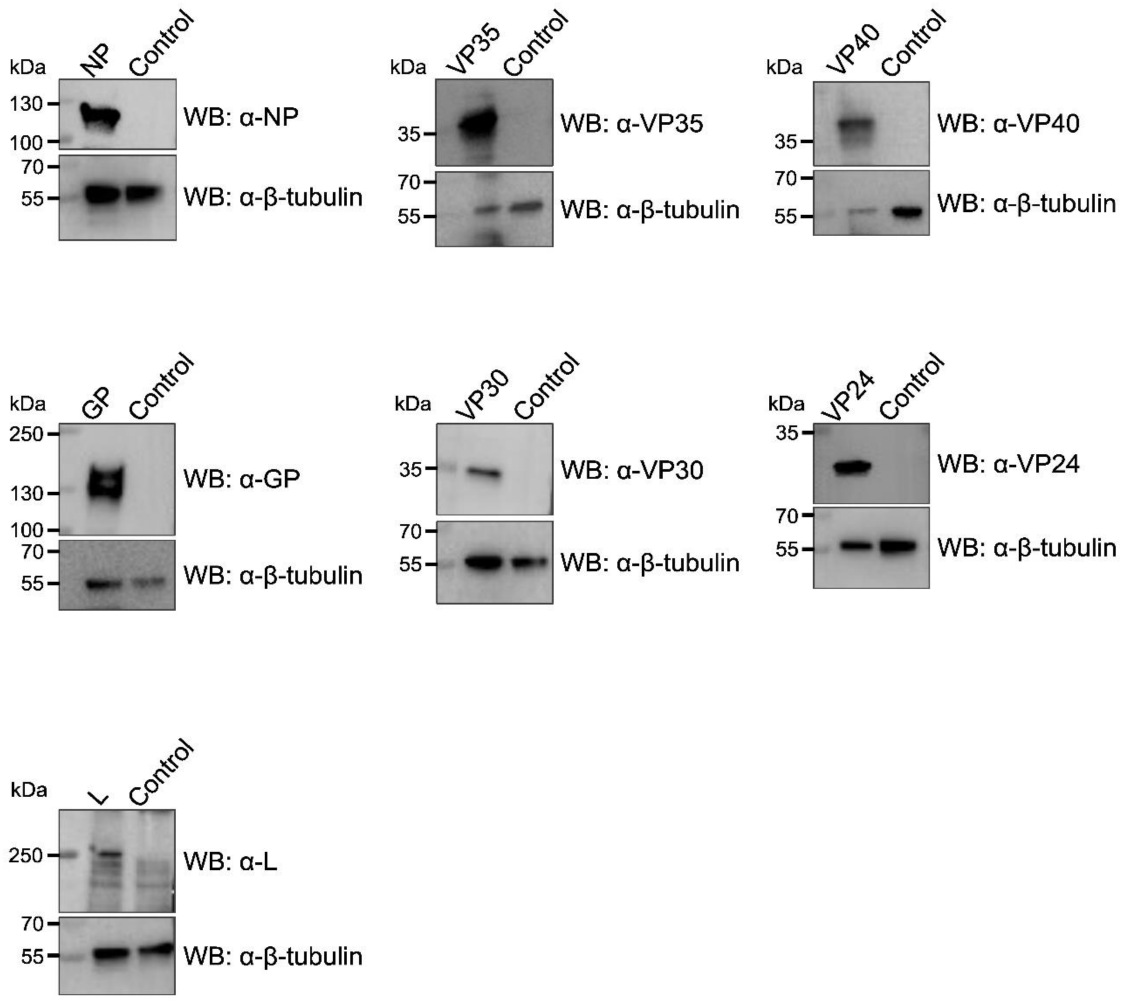

Supplementary Fig. 3: Evaluation of EBOV protein expression in plasmid transfected 293 cells at 48 hpt. 293 cells in 6-well plates were transfected with 2 μg of plasmid encoding each EBOV protein. For Western blotting, 10 μg of total protein were loaded for detection of NP, GP, VP30, VP24, and L, and 4.5 μg of total protein were loaded for detection of VP35 and VP40. Details of the primary and secondary antibodies are provided in the **SI Appendix, Table S2**. Note that expression levels should not be directly compared across proteins, as different antibodies with varying sensitivities were used for detection.

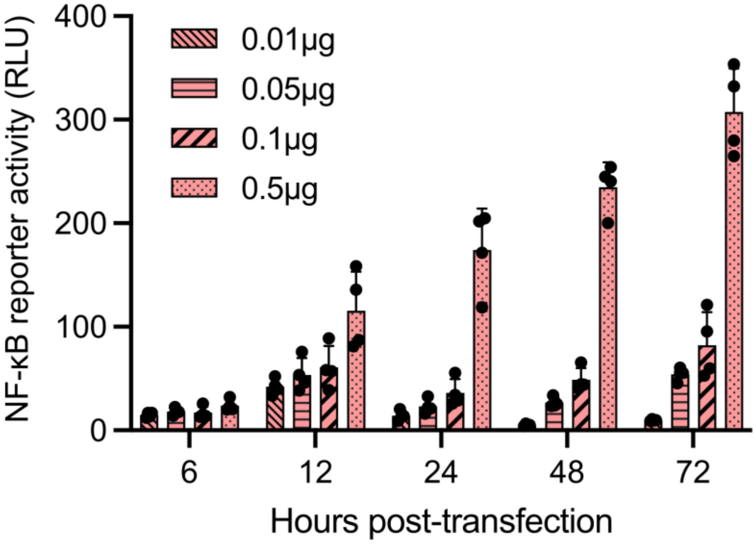

Supplementary Fig. 4: Time- and dose-kinetics of NF-κB-responsive reporter activity in 293 cells expressing EBOV VP40. Data are shown with mean ± SD (n = 3 independent experiments).

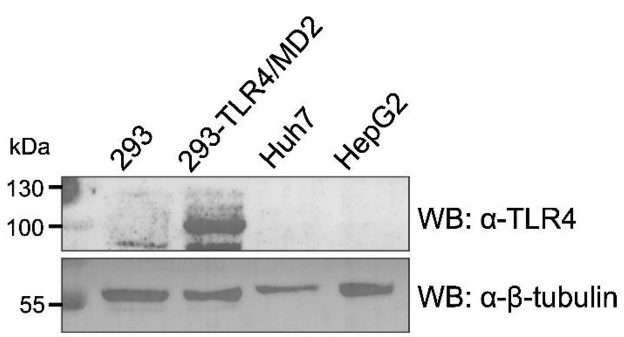

Supplementary Fig. 5: Evaluation of TLR4 expression in Huh7 and HepG2 cells. For Western blotting, 20 µg (293, 293-TLR4/MD2, or HepG2) or 37 µg (Huh7) of total protein were analyzed. Details of the primary and secondary antibodies are provided in the **SI Appendix, Table S2**.

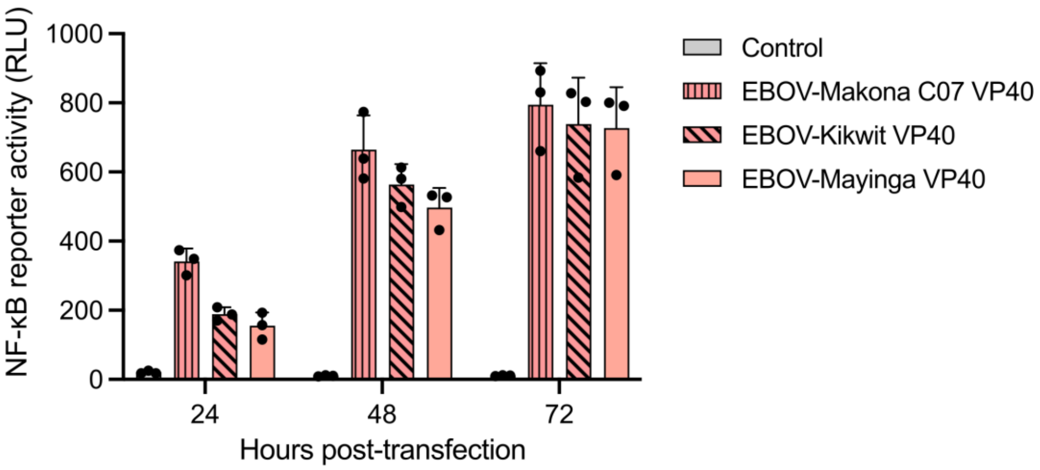

Supplementary Fig. 6: NF-κB-responsive luciferase reporter activity in 293 cells expressing VP40 derived from EBOV variant Mayinga, Kikwit, or Makona-07. Data are shown with mean ± SD (n = 3 independent experiments).

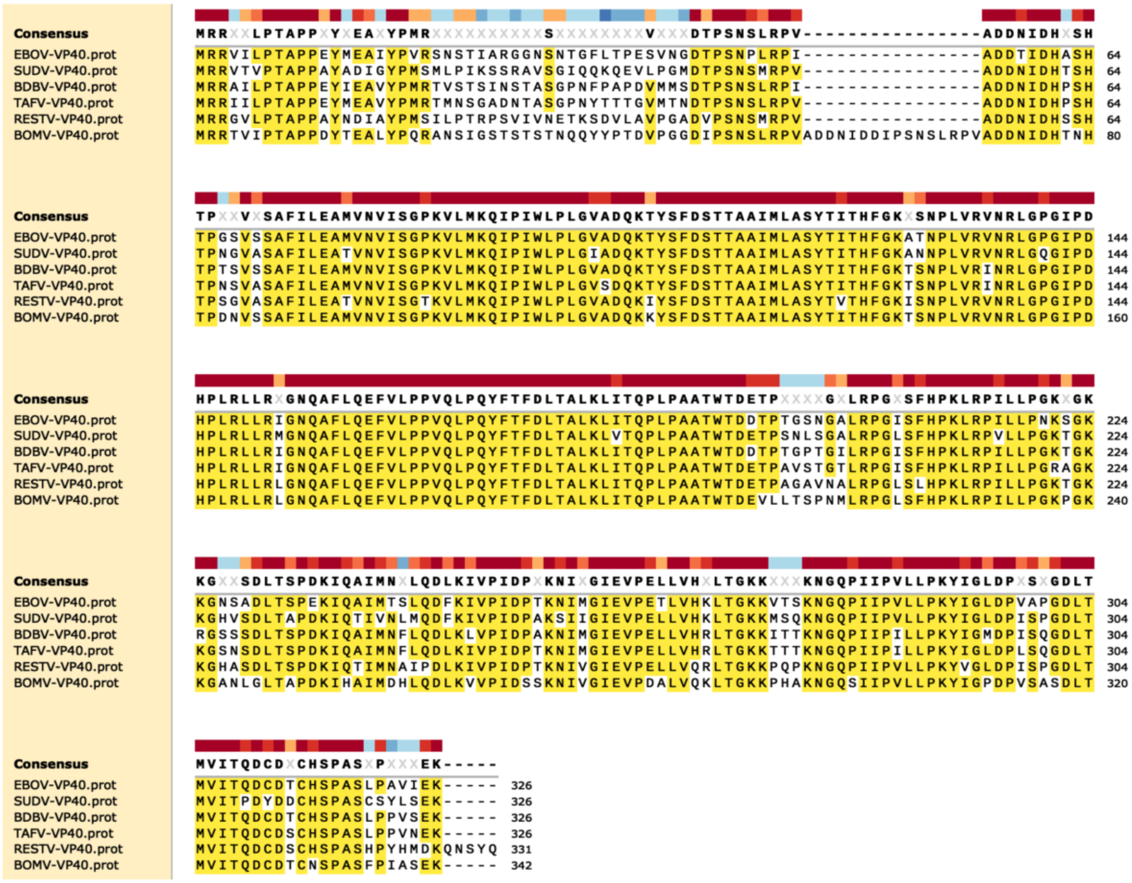

Supplementary Fig. 7: Amino acid sequence alignment of VP40 from six different ebolaviruses. Amino acid sequences were aligned using SnapGene version 7.2.1. Fully conserved amino acids across VP40 from six ebolaviruses are highlighted in yellow, with sequence conservation indicated by colored blocks.

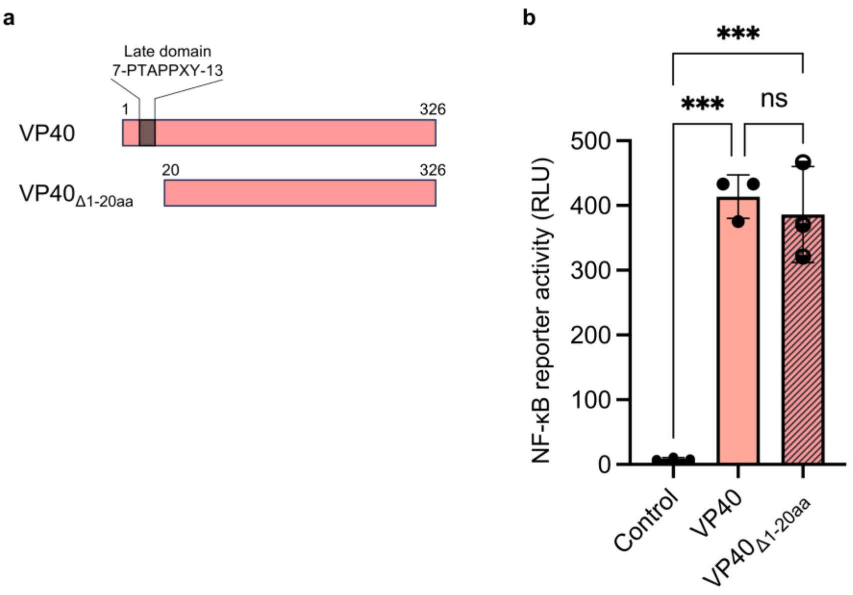

Supplementary Fig. 8: Examination of the importance of two classical late domains in VP40-mediated NF-κB activation. a Diagram of EBOV VP40 and a mutant with the first 20 amino acids deleted ($VP40_{\Delta 1\text{-}20aa}$). **b** NF-κB-responsive luciferase reporter activity in 293 cells expressing either EBOV VP40 or $VP40_{\Delta 1\text{-}20aa}$ at 72 hpt. Data are shown with mean ± SD (n = 3 independent experiments). ns > 0.05, ***$P \leq 0.001$; ordinary one-way ANOVA.

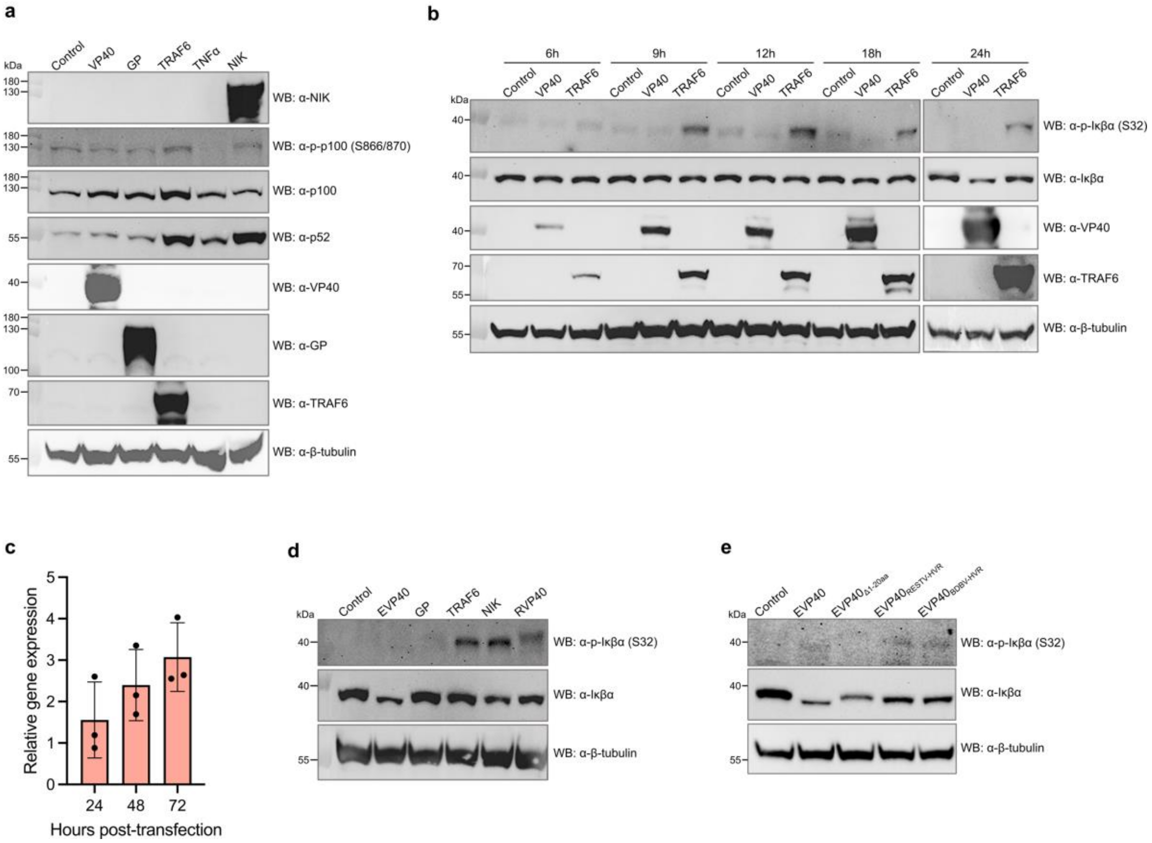

Supplementary Fig. 9: Examination of activation status of host proteins in NF-κB signaling pathways in the presence of EBOV VP40. a Western blotting for molecules involved in the non-canonical NF-κB pathway. 293 cells were transfected with plasmid EBOV VP40, EBOV GP, TRAF6, or NIK and harvested at 24 hpt or treated with 15 ng/ml of TNFα for 1 hour and then harvested. **b** Western blotting for phosphorylated-Iκβα or Iκβα in 293 cells expressing EBOV VP40 or TRAF6. **c** Quantification of Iκβα mRNA in EBOV VP40-expressing 293 cells. Extracted RNA was reverse transcribed using OligodT primer, and cDNA was used for qRT-PCR with iTaq Universal Probes Supermix (BioRad), Hs00355671_g1 NFKBIA (Thermo Scientific) and Hs00355671_g1 NFKBIA (Thermo Scientific). GAPDH was used as a reference gene (the primer/TaqMan probe sequences are shown in the **SI Appendix, Table S3)**. Delta-delta C_t values were used to determine their relative expression as fold changes. Details on RNA preparation are provided in the Materials and Methods section. **d** Western blotting for phosphorylated-Iκβα or Iκβα in 293 cells expressing EBOV VP40, EBOV GP, TRAF6, NIK or RESTV VP40 at 24 hpt. **e** Western blotting for phosphorylated-Iκβα or

Iκβα in 293 cells expressing EBOV VP40, EBOV $VP40_{\Delta 1\text{-}20aa}$, or chimeric VP40 proteins where the HVR of EBOV VP40 was replaced with that of RESTV or BDBV ($EVP40_{HVR\text{-}RESTV}$ and $EVP40_{HVR\text{-}BDBV}$) at 24 hpt. Control: Empty vector transfected. For **c**, data are shown with mean ± SD (n = 3 independent experiments).

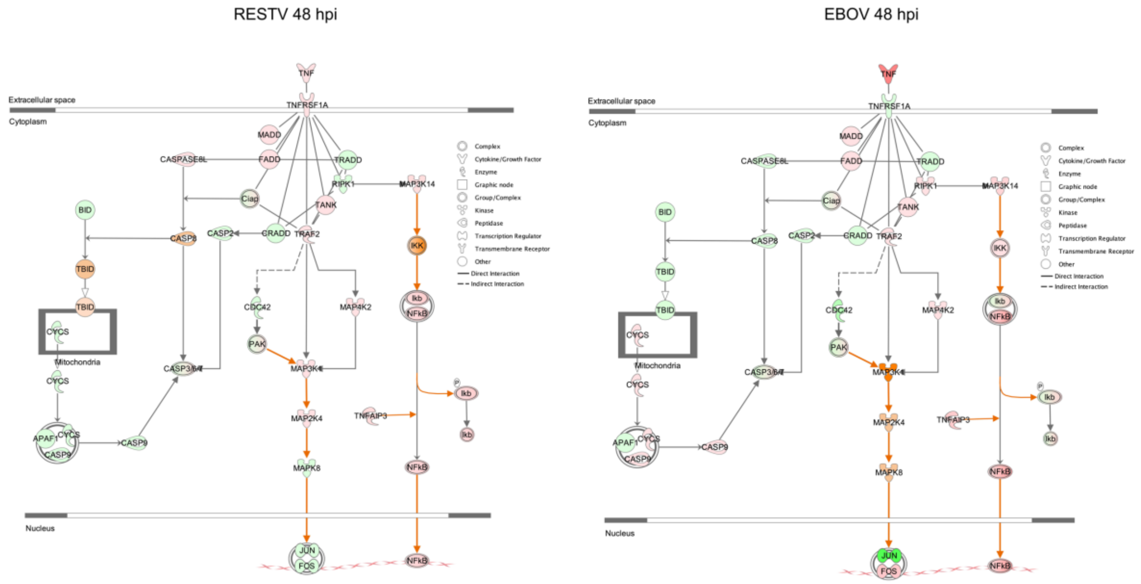

Supplementary Fig. 10: IPA TNFR1 Signaling Canonical Pathway overlaid with RNA-seq gene expression data from EBOV- or RESTV-infected 293 cells. Red and green shadings indicate positive fold change (upregulation) and negative fold change (downregulation) relative to the time-matched mock-infected controls, respectively. Orange shading indicates predicted activation. Darker red or green shading indicates that genes met DE criteria (fold change > |1.5|, adjusted *P*-value < 0.05), while lighter shading indicates that they did not. Darker orange shading indicates a higher z-score. Orange and blue lines indicate predicted activation and inhibition, respectively. Gray shading indicates insufficient information to predict molecular activity. Solid lines indicate known direct interactions. Arrowheads or line caps indicate directionality.

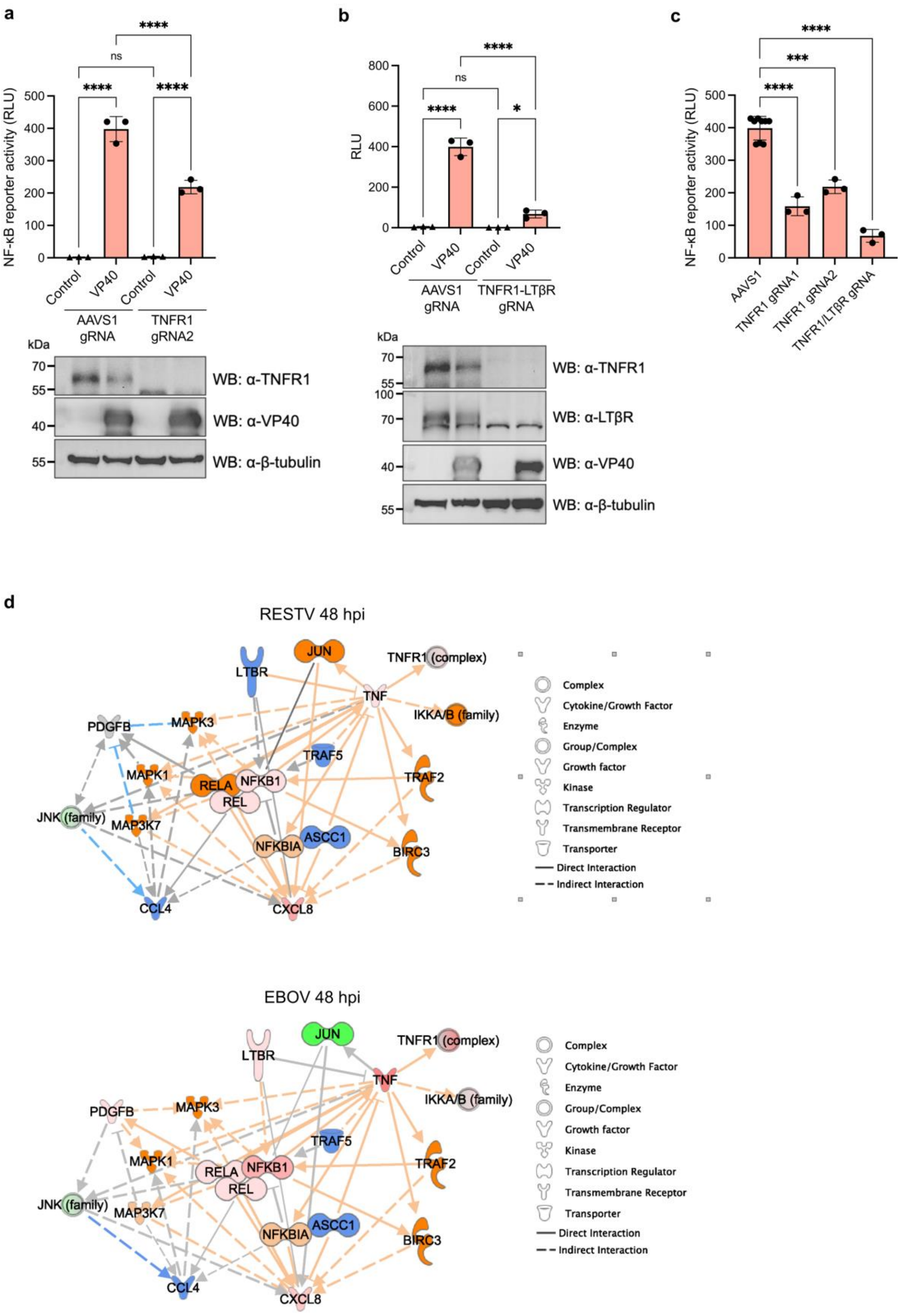

a
NF-κB reporter activity (RLU)
Control
VP40
AAVS1 gRNA
TNFR1 gRNA2
ns
****
WB: α-TNFR1
WB: α-VP40
WB: α-β-tubulin
kDa
b
RLU
AAVS1 gRNA
TNFR1-LTβR gRNA
*
WB: α-LTβR
c
AAVS1
TNFR1 gRNA1
TNFR1 gRNA2
TNFR1/LTβR gRNA
***
d
RESTV 48 hpi
EBOV 48 hpi
JUN
LTBR
TNFR1 (complex)
TNF
IKKA/B (family)
PDGFB
MAPK3
TRAF5
MAPK1
RELA
NFKB1
REL
TRAF2
JNK (family)
MAP3K7
NFKBIA
ASCC1
BIRC3
CCL4
CXCL8
Complex
Cytokine/Growth Factor
Enzyme
Group/Complex
Growth factor
Kinase
Transcription Regulator
Transmembrane Receptor
Transporter
Direct Interaction
Indirect Interaction

Supplementary Fig. 11: Assessment of contribution of TNFR1 to NF-κB activation induced by EBOV VP40. a NF-κB-responsive reporter activity in TNFR1-knockout (gRNA 2) 293 cells expressing EBOV VP40 at 48 hpt. **b** NF-κB-responsive luciferase reporter activity in TNFR1/LTβR-knockout 293 cells expressing EBOV VP40 at 48 hpt. **c** Summary of NF-κB-responsive reporter activity in TNFR1 or TNFR1/LTβR-knockout 293 cells expressing EBOV VP40 at 48 hpt. **d** IPA custom network generated with RNA-seq result from EBOV- or RESTV-infected 293 cells. Molecules associated with EBOV VP40-mediated inflammation-associated genes were selected to generate networks. NF-κB subunits (REL, RELA, NFKBI) are clustered at the network center as they directly interact and form a complex. Red and green shadings indicate upregulated and downregulated genes meeting DE criteria, respectively (fold change > |1.5|, adjusted *P*-value < 0.05). Orange and blue shadings indicate predicted activation and inhibition, respectively. Gray shading indicates insufficient information to predict molecular activity. Solid and dashed lines indicate known direct interactions and indirect interactions, respectively. Arrowheads or line caps indicate directionality. Control: Empty vector transfected. For **a**-**c**, data are shown with mean ± SD (n = 3 independent experiments). ns > 0.05, *$P \leq 0.05$, ***$P \leq 0.001$, ****$P \leq 0.0001$; ordinary one-way ANOVA.

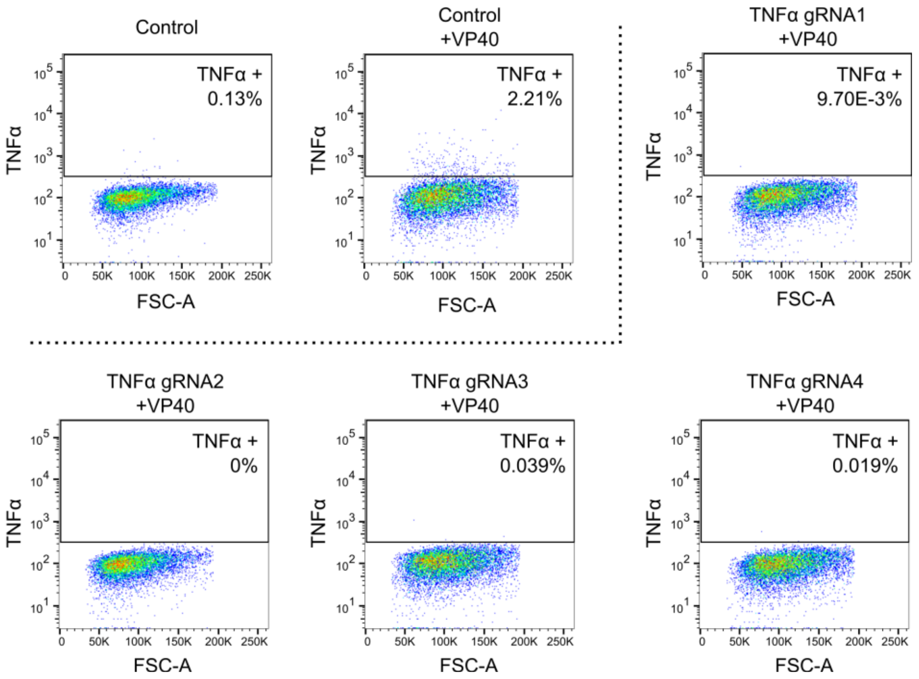

Supplementary Fig. 12: Quantification of intracellular TNFα in TNFα-knockout 293 cells expressing EBOV VP40 by flow cytometry. Control: CRISPR-Cas9 targeting AAVS1. 293 cells or TNFα knockout 293 cells (3.5 x 10^5 cells) were seeded in 6-well plate 1 day before transfection and were transfected with a pCAGGs-EBOV VP40 (2 µg). At 72 hpt, cells were treated with BD GolgiPlug Protein Transport Inhibitor (Brefeldin A) (BD Biosciences) at a final concentration of 1:1000 for 6 hours followed by fixation with 2% paraformaldehyde for 30 min. Cells were permeabilized with Intracellular Staining Permeabilization Wash Buffer (BioLegend) and then stained with 3 µl of PE-anti human TNFα antibody (BioLegend) in a 100 µl of volume for 30 min. The samples were measured using BD LSR II Flow Cytometer. All flow cytometry data were analyzed using Flow Jo software.

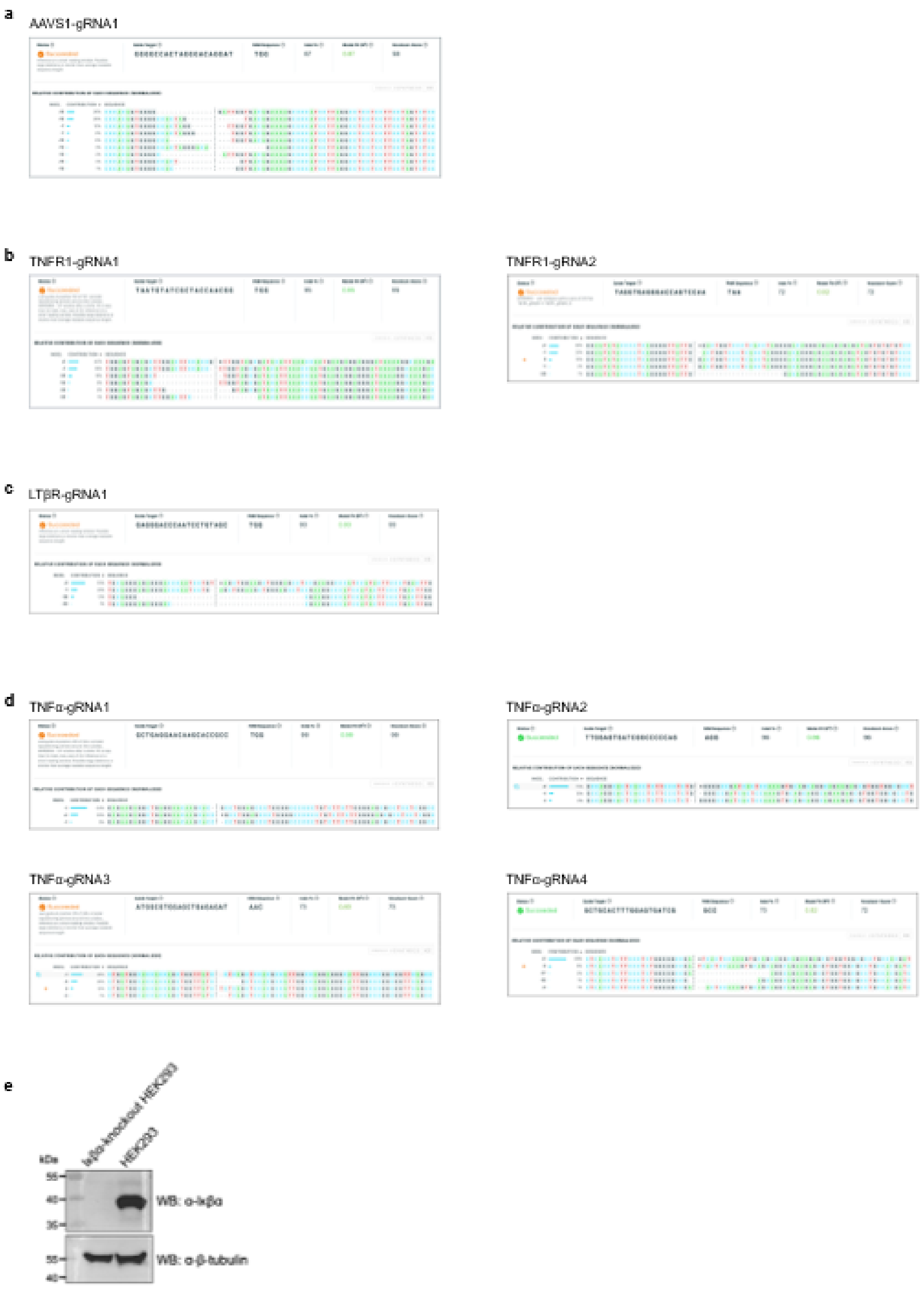

Supplementary Fig. 13: Confirmation of CRISPR editing. a Synthego ICE analysis

for CRISPR editing targeting **a** AAVS1, **b** TNFR1, **c** LTβR, **d** TNFα. **e** Western blotting for Iκβα in Iκβα-knockout 293 and wild-type 293 cells.

Supplementary Table 1. Sequences of gRNA

Target	No.	Sequence
AAVS1	gRNA1	GGGGCCACTAGGGACAGGAT
Iκβα	gRNA1	GGTTGGTGATCACAGCCAAG
TNFR1	gRNA1	TAATGTATCGCTACCAACGG
	gRNA2	TTGGACTGGTCCCTCACCTA
LTβR	gRNA1	GAGGGACCCAATCCTGTAGC
TNFα	gRNA1	GCTGAGGAACAAGCACCGCC
	gRNA2	TTGGAGTGATCGGCCCCCAG
	gRNA3	CGATCACTCCAAAGTGCAGC
	gRNA4	ATCTCTCAGCTCCACGCCAT

Supplementary Table 2. List of primary and secondary antibodies used for western blotting in this study

Target protein	Company	Catalog number	Dilution
EBOV VP40	IBT BioServices	0201-017	1:2000
EBOV GP	IBT BioServices	0301-015	1:1000
EBOV NP	Biomatik	N/A[*1]	1:1000
EBOV VP35	Invitrogen	PA5-112029	1:1000
EBOV VP30	Novus Biologicals	NBP3-13345	1:1000
EBOV VP24	N/A[*2]	N/A	1:1000
EBOV L	IBT BioServices	0301-045	1:1000
FLAG	MilliporeSigma	F7425	1:2000
β-tubulin	Abcam	ab6046	1:2000

TRAF6	Cell Signaling Technology	8028	1:1000
LaminA/C	Cell Signaling Technology	4777	1:1000
GAPDH	Santa Cruz Biotechmology	sc-47724	1:500
TLR4	Cell Signaling Technology	Abcam	1:1000
p65	Cell Signaling Technology	4764	1:1000
RelB	Cell Signaling Technology	10544	1:1000
c-Rel	Cell Signaling Technology	12707	1:1000
NF-κB1 p105/p50	Cell Signaling Technology	3035	1:1000
NF-κB2 p100/p52	Cell Signaling Technology	4882	1:1000
p-NF-κB2 p100	Cell Signaling Technology	4810	1:1000
NIK	Cell Signaling Technology	4994	1:1000
p-IKKα/β (Ser176/180)	Cell Signaling Technology	2697	1:1000
IKKα	Cell Signaling Technology	11930	1:1000
IKKβ	Cell Signaling Technology	8943	1:1000
p-Iκβα (Ser32)	Cell Signaling Technology	2859	1:1000
Iκβα	Cell Signaling Technology	4814	1:1000
TNF-R1	Cell Signaling Technology	3736	1:1000
LTβR	Proteintech	20331-1-AP	1:1000
Anti-mouse-HRP	Jackson ImmunoResearch Laboratories	715-035-150	1:25000
Anti-rabbit-HRP	Jackson ImmunoResearch Laboratories	715-035-152	1:25000

[*1] Affinity purified rabbit polyclonal antibody was produced against peptide antigen Cys-PAVSSGKNIKRT from EBOV Mayinga NP protein by Biomatik and used as a primary antibody.

[*2] Rabbit anti-VP24 was generated by immunizing rabbit with purified VP24 peptide corresponding to amino acids 5-20 (2).

Supplementary Table 3. Sequences of TaqMan primers and primers

Target	Probe/Primer	Sequence
IL-8	Probe	**6FAM-**TggCgCAgTgTggTCCACTC**--BBQ**
	Primer (Fwd)	gCTCTgTgTgAAggTgCAgTT
	Primer (Rev)	AgCTCTCTTCCATCAgAAAgC
TNF-α	Probe	**6FAM-**TCgTggCAggCgCCACC**--BBQ**
	Primer (Fwd)	gCggTgCTTgTTCCTCA
	Primer (Rev)	ggCCAgAgggCTgATTAg
MIP-1β	Probe	**6FAM-**TCCTCgTgCTAgTAgCTgCCTTCTgC**--BBQ**
	Primer (Fwd)	ACCATgAAgCTCTgCgTgA
	Primer (Rev)	gAggAAgCTTCCTCgCg
GAPDH	Probe	**YAK-**CAAgCTTCCCgTTCTCAgCCT**--BBQ**
	Primer (Fwd)	gAAggTgAAggTCggAgTC
	Primer (Rev)	gAAgATggTgATgggATTTC

SI References

1. Y. Tsuda *et al.*, An Improved Reverse Genetics System to Overcome Cell-Type-Dependent Ebola Virus Genome Plasticity. *J Infect Dis* **212 Suppl 2**, S129-137 (2015).
2. L. Banadyga *et al.*, Ebola virus VP24 interacts with NP to facilitate nucleocapsid assembly and genome packaging. *Sci Rep* **7**, 7698 (2017).
3. H. Niwa, K. Yamamura, J. Miyazaki, Efficient selection for high-expression transfectants with a novel eukaryotic vector. *Gene* **108**, 193-199 (1991).
4. B. N. Zell *et al.*, Development of a Pentacistronic Ebola Virus Minigenome System. *Viruses* **17** (2025).
5. F. Mercurio *et al.*, IKK-1 and IKK-2: cytokine-activated IkappaB kinases essential for NF-kappaB activation. *Science* **278**, 860-866 (1997).
6. S. Sun, J. Elwood, W. C. Greene, Both amino- and carboxyl-terminal sequences within I kappa B alpha regulate its inducible degradation. *Mol Cell Biol* **16**, 1058-1065 (1996).
7. N. E. Sanjana, O. Shalem, F. Zhang, Improved vectors and genome-wide libraries for CRISPR screening. *Nat Methods* **11**, 783-784 (2014).
8. B. Institute (Babraham Bioinformatics.
9. V. A. Schneider *et al.*, Evaluation of GRCh38 and de novo haploid genome assemblies demonstrates the enduring quality of the reference assembly. *Genome Res* **27**, 849-864 (2017).
10. A. Dobin *et al.*, STAR: ultrafast universal RNA-seq aligner. *Bioinformatics* **29**, 15-21 (2013).
11. Y. Liao, G. K. Smyth, W. Shi, The R package Rsubread is easier, faster, cheaper and better for alignment and quantification of RNA sequencing reads. *Nucleic Acids Res* **47**, e47 (2019).
12. M. I. Love, W. Huber, S. Anders, Moderated estimation of fold change and dispersion for RNA-seq data with DESeq2. *Genome Biol* **15**, 550 (2014).
13. A. Subramanian *et al.*, Gene set enrichment analysis: a knowledge-based approach for interpreting genome-wide expression profiles. *Proc Natl Acad Sci U S A* **102**, 15545-15550 (2005).
14. G. Yu, L. G. Wang, Y. Han, Q. Y. He, clusterProfiler: an R package for comparing biological themes among gene clusters. *Omics* **16**, 284-287 (2012).
15. R. C. T. (2025) (2025) R: A Language and Environment for Statistical Computing. (R Foundation for Statistical Computing, Vienna, Austria).
16. C.-H. G. Guangchuang Yu (2025) enrichplot: Visualization of Functional Enrichment Result. (https://yulab-smu.top/biomedical-knowledge-mining-book/).
17. K. R (2018) pheatmap: Pretty Heatmaps. (https://github.com/raivokolde/pheatmap).
18. W. H (2016) ggplot2: Elegant Graphics for Data Analysis. (Springer-Verlag New York, https://ggplot2.tidyverse.org).
